# Including Fibroblast Growth Factor-21 in Combined Biomarker Panels Improves Predictions of Liver Steatosis Severity in Children

**DOI:** 10.3389/fped.2019.00420

**Published:** 2019-10-30

**Authors:** Man-Chin Hua, Jing-Long Huang, Ching-Chih Hu, Tsung-Chieh Yao, Ming-Wei Lai

**Affiliations:** ^1^Department of Pediatrics, Chang Gung Memorial Hospital, Keelung, Taiwan; ^2^Chang Gung University College of Medicine, Taoyuan, Taiwan; ^3^Division of Allergy, Asthma and Rheumatology, Department of Pediatrics, Chang Gung Memorial Hospital, Taoyuan, Taiwan; ^4^Division of Hepatology, Department of Hepatogastroenterology, Chang Gung Memorial Hospital, Keelung, Taiwan; ^5^Division of Gastroenterology, Department of Pediatrics, Chang Gung Memorial Hospital, Taoyuan, Taiwan; ^6^Liver Research Center, Chang Gung Memorial Hospital, Taoyuan, Taiwan

**Keywords:** FGF-21, biochemical parameters, BMI, combined analysis, predictors, high-grade liver steatosis, liver ultrasound, childhood obesity

## Abstract

**Background:** Previous studies reported conflicting results regarding the association between fibroblast growth factor-21 (FGF-21) and non-alcoholic fatty liver disease (NAFLD). This study aimed to evaluate the feasibility of combining FGF-21, obesity indices, and biochemical tests for predicting high-grade liver steatosis in children.

**Methods:** A total of 203 children and adolescents aged 5–18 years were enrolled, and their anthropometric data, body composition, liver ultrasound score for NAFLD (range, 0–6), biochemical test results, and FGF-21, leptin, and adiponectin levels were analyzed. Children were categorized according to body mass index (BMI) and NAFLD scores. Univariate analysis and multivariate linear regression were used to identify independent predictors for the degree of liver steatosis. The accuracy of the models was also evaluated using a receiver-operating characteristic (ROC) curve.

**Results:** FGF-21 levels were significantly higher in subjects with high-grade liver steatosis (*P* < 0.001). In obese and overweight children, regression analysis indicated that higher BMI and higher gamma-glutamyl transferase (γ-GT), triglycerides (TG), and FGF-21 levels were independent risk factors strongly correlated with NAFLD scores. FGF-21 combined with any of the above parameters showed a larger area under the ROC (AUROC, 0.861–0.873) than either parameter used alone. Overall, the best performance was obtained by combing FGF-21, γ-GT, and TG, with an AUROC of 0.871, specificity of 82.54%, and sensitivity of 83.78% for predicting high-grade liver steatosis.

**Conclusion:** BMI, FGF-21, γ-GT, and TG levels were strongly correlated with liver steatosis severity. Including FGF-21 in the biomarker panels may improve the accuracy for identifying obese and overweight children with high-grade liver steatosis.

## Introduction

Concomitant with the rising prevalence of childhood obesity, non-alcoholic fatty liver disease (NAFLD) has been increasingly recognized in children over recent decades (1). NAFLD may progress from steatosis to non-alcoholic steatohepatitis (NASH), fibrosis, and cirrhosis, which may result in liver failure or hepatocellular carcinoma in the long run (2, 3). Hence, we must address the silent health threats and need for monitoring of the target group. Although liver histology is the gold standard for differentiating NAFLD from the normal healthy group, it is invasive and impractical for screening purposes. Some studies have surveyed non-invasive parameters including anthropometric measurements (e.g., body mass index [BMI] and waist circumference) (4–6), body fat distribution (e.g., visceral and subcutaneous fat) (7, 8), biochemical data (e.g., homeostasis model of assessment values for insulin resistance [HOMA-IR] and hypertriglyceridemia), and adipokine patterns (e.g., leptin and adiponectin) in NAFLD patients (9–12). However, the ideal parameters to differentiate NAFLD from the obese but healthy group or simple steatosis from NASH are still lacking.

Fibroblast growth factor-21 (FGF-21), which is mainly secreted by the liver, adipose tissue, skeletal muscle, and pancreas, has recently been identified to affect glucose and lipid metabolism in hepatocytes and adipocytes (13, 14). Some studies reported that FGF-21 protects against hepatic steatosis and damage (14), whereas others presented conflicting results (15–17); thus, the role of FGF-21 on NAFLD requires further investigation.

The present study involved a thorough evaluation of anthropometric data and body composition using different techniques and an examination of the correlation between biochemical markers, such as adipokines and FGF-21 levels, and liver steatosis severity. Here we aimed to explore which parameters were independent predictors for liver steatosis and evaluate the feasibility of combining indices to predict high-grade liver steatosis in overweight and obese children.

## Methods

### Study Subjects

From March 2015 to August 2017, we recruited obese and overweight children aged 5–18 years who were willing to undergo anthropometric and body composition measurements, blood sampling, and fatty liver sonographic screening at the outpatient clinic of Chang Gung Memorial Hospital, Keelung, and healthy controls from the Prediction of Allergies in Taiwanese Children (PATCH) cohort study (18). Obese children and adolescents with abnormal aminotransferase levels referred to our pediatric gastroenterology outpatient clinic were also recruited. All of the parents and children received verbal and written information about the study's design and objectives. All participants were enrolled after written informed consent was obtained from their parents. Those subjects who received medications that could affect glucose metabolism and those who presented chronic liver diseases, including hepatitis B, hepatitis C, autoimmune hepatitis, and Wilson's disease, were excluded. This study was approved by the Research Ethics Committee of Chang Gung Memorial Hospital (104-7100C, 106-3610C, and 104-7145B) and complied with the Declaration of Helsinki.

### Anthropometric and Body Composition Measurements

Body weight (BW) and body height (BH) were measured using an electronic scale (Super-view-HW3050; Hualien, Taiwan; precisions of 0.1 kg and 0.1 cm, respectively) with the participants wearing light clothes and no shoes. BH and BW were used to calculate BMI in kg/m^2^. Children with a BMI ≥ 95th percentile were defined as obese, those with a BMI ≥ 85–95th percentile were defined as overweight, and those with a BMI = 10–85th percentile were defined as normal. The age- and sex-specific standards were based on the BMI charts officially declared by Department of Health in Taiwan (19). Waist circumference was measured at the level of the umbilicus. Hip circumference was measured at the point of maximal protrusion of the buttocks. The waist-to-hip and waist-to-height ratios were calculated from these measurements. Two skinfold sites on the bilateral triceps and gastrocnemius were measured with a skinfold caliper (Slim guide, Michigan, USA) to the nearest 0.5 mm using standard procedures. Mean skinfold thickness values were calculated from skinfold measurements of the bilateral triceps and gastrocnemius.

Body composition was measured using a multi-frequency (20 and 100 kHz) bioelectrical impedance analysis (BIA) device that uses an 8-point tactile electrode system (InBody 230, Seoul, Korea) to yield detailed data on body composition, including body fat percentage, trunk fat percentage, total body fat, fat-free mass, total body water, and total body muscle (20). The InBody 230 is suitable for individuals aged 3–99 years. All anthropometric measurements and BIA were performed by two well-trained research assistants.

### Liver Ultrasonographic Examination and Scoring System for Liver Steatosis

Liver steatosis severity was determined in each participant using ultrasonography by the same pediatric gastroenterologist (21). Liver steatosis severity was assessed on ultrasonography using three scoring items that have been described by Hamaguchi et al. (22). This score is referred to as the NAFLD score in this study and is composed of: *(1) Bright liver and hepatorenal echo contrast (score 0-3)*. Score 0: both liver echogenicity and hepatorenal contrast was normal; Score 1: either liver echogenicity or hepatorenal contrast was increased; Score 2: both liver echogenicity and hepatorenal contrast were mildly increased; Score 3: both liver echogenicity and hepatorenal contrast were significantly increased; *(2) Deep attenuation of the diaphragm (score 0-2)*. Score 0: the diaphragm could be clearly distinguished by an observer; Score 1: visualization of the diaphragm was obscure, but an observer could distinguish the diaphragm; Score 2: an observer could not distinguish the diaphragm *(3) Visualization of the intrahepatic vessels (score, 0-1)*. Score 0: no evidence of vessel blurring; Score 1: the border of intrahepatic vessels were unclear and the lumen of intrahepatic vessels were narrowed. Standardized views of the liver were obtained to enable scoring of these three items (NAFLD score 0–6) (22). If the score for the hepatorenal echo contrast and bright liver was ≥1, we summed up all scores and defined the subjects as having liver steatosis. If hepatorenal echo contrast and bright liver scores were zero, the total score was defined as zero (22).

### Collection of Biochemical Data

Blood samples (10-mL aliquots) were collected in test tubes containing ethylenediaminetetraacetic acid (1g/L) after the subjects fasted overnight, and the following markers were analyzed: alanine aminotransferase (ALT), aspartate aminotransferase (AST), gamma-glutamyl transferase (γ-GT), glucose, insulin, triglyceride (TG), total cholesterol, low-density lipoprotein cholesterol (LDL-C), and high-density lipoprotein cholesterol (HDL-C). HOMA-IR was also obtained (23). The diagnoses of hepatitis B and C, autoimmune hepatitis, and Wilson's disease were excluded using the appropriate diagnostic tests.

### Measurement of FGF-21, Adiponectin, and Leptin

Plasma was separated by centrifugation (3,000 rpm for 10 min) at room temperature within 3 h of blood collection and stored at −80°C until use. After the samples were thawed, the concentrations of FGF-21 (pg/mL), adiponectin (ng/mL), and leptin (pg/mL) were determined using enzyme-linked immunosorbent assay kits (R&D Systems, Minneapolis, MN, USA) according to the manufacturer's instructions. The assays had a minimum detection limit of 8.69 pg/mL for FGF-21, 0.891 ng/mL for adiponectin, and 7.8 pg/mL for leptin.

### Group Definitions

#### Obese Children With Liver Steatosis

Obese (BMI ≥ 95th percentile) or overweight (BMI ≥ 85–95th percentile) children with an NAFLD score ≥ 1. Specifically, an NAFLD score of 4–6 was defined as *high-grade liver steatosis*, while an NAFLD score of 1–3 was defined as *low-grade liver steatosis*.

#### Simple Obese Children

Obese (BMI ≥ 95th percentile) or overweight (BMI ≥ 85–95th percentile) children with an NAFLD score equal to 0.

#### Healthy Controls

Healthy children with a normal BMI (BMI = 10–84th percentile), NAFLD score = 0, and no medical disorders.

### Statistical Analysis

Continuous and normally distributed variables are expressed as mean ± SD and were analyzed using one-way analysis of variance. For continuous variables with a non-normal distribution, the differences between two study groups were estimated using the Mann-Whitney *U* test. In obese and overweight children, the relationship between NAFLD score and predictive parameters, such as obesity indices (e.g., BMI, waist-to-hip ratio, waist-to-height ratios, mean skinfold thickness, and body fat composition), biochemical data, and the logarithmically transformed concentrations of FGF-21, adiponectin, and leptin were determined using a univariate linear regression model. The parameters with values of *P* < 0.001 on univariate logistic regression analysis were entered in the stepwise linear regression model. Finally, the accuracy of the models was evaluated using the area under a receiver operating characteristic (AUROC) curve with 95% confidence interval (95% CI) and the sensitivity and specificity of the final model were calculated. For combined analysis with normally and non-normally distributed variables, all values in the ROC models were logarithmically transformed in the statistics. Two-tailed *P*-values < 0.05 were considered significant. The statistical analysis was performed using SPSS Statistics version 20 (IBM, Armonk, NY, USA).

## Results

### Subject Characteristics

A total of 203 children and adolescents (102 male, 101 female; mean age, 13.69 ± 3.16 years) were enrolled and divided into healthy controls (*n* = 89), simple obese (*n* = 31), and obese with liver steatosis (*n* = 83) groups. There were no intergroup differences in mean age or sex. Eighty-three of the 114 (72.8%) obese or overweight children presented with liver steatosis, of whom 42 (50.6%) were defined as having low-grade steatosis and 41 (49.4%) were defined as having high-grade liver steatosis.

### Intergroup Comparison of Obesity Indices and Biochemical Data

As shown in Table 1, a significant and progressive increase in BMI, waist circumference, waist-to-hip ratio, and waist-to-height ratio was found from the healthy control to the simple obese to the obese with steatosis groups (*P* < 0.001). With regard to body composition data measured by BIA, body fat percentage, trunk fat percentage, and total body fat weight were significantly different across the three groups (*P* < 0.001). Moreover, the HOMA-IR index, AST, ALT, γ-GT, and TG levels were significantly higher, whereas the HDL-C levels were significantly lower in obese children with liver steatosis compared with the healthy controls and simple obese children (*P* < 0.001) (Table 1).

**Table 1:** Comparison of clinical and laboratory parameters in healthy controls, simple obese children, and obese children with liver steatosis.

	**Healthy controls (*N* = 89)**	**Simple obese children (*N* = 31)**	**Obese children with liver steatosis (*N* = 83)**
**BASIC AND ANTHROPOMETRIC DATA**			
Age (y)	14.06 ± 3.97	13.74 ± 3.60	12.78 ± 3.27
Gender, male (%)	43 (48.3)	13 (41.9)	46 (55.4)
BMI (kg/m^2^)	18.69 ± 2.21	24.54 ± 3.54^^a^^	27.30 ± 3.53^^a^^b^^
Waist circumference (cm)	66.72 ± 8.43	80.76 ± 11.91^^a^^	88.40 ± 14.83^^a^^b^^
Hip circumference (cm)	85.10 ± 11.32	97.03 ± 12.65^^a^^	99.00 ± 14.08^^a^^
Waist-hip ratio	0.79 ± 0.06	0.83 ± 0.07^^a^^	0.90 ± 0.12^^a^^b^^
Waist-height ratio	0.43 ± 0.04	0.51 ± 0.05^^a^^	0.57 ± 0.09^^a^^b^^
Mean skinfold thickness (cm)	1.52 ± 1.53	1.82 ± 0.70	2.02 ± 1.32^^a^^
**DATA FROM BIA**			
Body fat (%)	20.83 ± 7.50	31.56 ± 8.06^^a^^	37.27 ± 7.31^^a^^b^^
Trunk fat (%)	18.63 ± 9.57	32.20 ± 8.58^^a^^	38.09 ± 7.10^^a^^b^^
Total body fat (kg)	10.17 ± 5.98	20.03 ± 7.40^^a^^	25.12 ± 7.35^^a^^b^^
Fat-free mass (kg)	36.34 ± 11.58	42.51 ± 13.20^^a^^	41.97 ± 11.57^^a^^
Total body water (kg)	26.65 ± 8.51	31.15 ± 9.64^^a^^	30.73 ± 8.42^^a^^
Total body muscle (kg)	19.56 ± 6.98	23.25 ± 7.95^^a^^	23.00 ± 7.02^^a^^
**BIOCHEMISTRY DATA**			
Fasting blood glucose (mg/dL)	86.21 ± 17.37	89.36 ± 5.08	90.69 ± 7.37
Insulin (μlU/ml)	4.92 ± 3.62	8.35 ± 5.72	14.87 ± 10.27^^a^^
HOMA-IR	1.39 ± 0.69	2.57 ± 1.26	3.69 ± 2.88^^a^^b^^
ALT (U/L)	14.07 ± 6.86	17.41 ± 11.03	50.18 ± 15.94^^a^^b^^
AST (U/L)	18.69 ± 6.82	18.04 ± 4.78	33.77 ± 23.32^^a^^b^^
γ-GT (U/L)	13.72 ± 5.68	15.17 ± 4.72	24.78 ± 12.63^^a^^b^^
Triglycerides (mg/dL)	72.01 ± 62.47	80.52 ± 40.29	117.76 ± 60.2^^a^^b^^
Total cholesterol (mg/dL)	167.97 ± 34.33	162.78 ± 32.55	176.21 ± 26.10
HDL-C (mg/dL)	55.28 ± 15.71	55.32 ± 14.04	44.75 ± 8.00^^a^^b^^
LDL-C (mg/dL)	94.27 ± 25.80	101.92 ± 27.78	106.65 ± 23.73

*P-value based on ANOVA test with multiple comparison analysis*.

a *Indicated significant differences (P-values <0.05) between liver steatosis group (or simple obese group) and healthy control group*.

b *Indicated significant differences (P-values < 0.05) between liver steatosis group and simple obese group*.

### Intergroup Comparison of FGF-21, Adiponectin, and Leptin Levels

**Figure 1:**
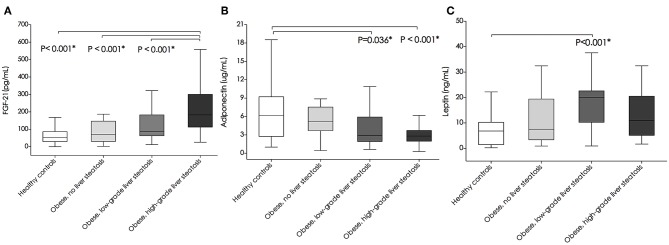
Comparison of plasma **(A)** fibroblast growth factor-21(FGF-21) levels (pg/mL), **(B)** adiponectin levels (ug/mL), and **(C)** leptin levels (ng/mL) between healthy controls, simple obese, and obese children with low-grade and high-grade liver steatosis. Box plots are shown as medians along with interquartile ranges. The differences between two study groups were estimated using Mann-Whitney *U* test. *P-values < 0.05 were considered statistically significant.

On the Mann-Whitney U-test, FGF-21 levels were significantly higher in the high-grade liver steatosis group than in the low-grade liver steatosis group, simple obese children, and healthy controls (median [IQR], 183.17 [113.03–301.14] pg/mL vs. 86.58 [62.76–183.02] pg/mL vs. 79.76 [30.91–146.76] pg/mL vs. 53.45 [29.78–87.21] pg/mL, *P* < 0.001, respectively) (Figure 1A). Adiponectin levels tended to decrease as the hepatic fat content was increased. Adiponectin levels were significantly lower in the low- and high-grade liver steatosis groups than in the healthy controls (median [IQR], 2.86 [1.93–5.92] μg/mL vs. 6.15 [2.75–9.22] μg/mL, *P* = 0.036; and 2.81 [1.98–3.72] μg/mL vs. 6.15 [2.75–9.22] μg/mL, *P* < 0.001, respectively) (Figure 1B). In contrast, the leptin levels in the study subjects were variable. Compared to those in the healthy controls, the leptin levels were significantly higher in the low-grade liver steatosis group (median [IQR], 20.06 [10.26–22.61] ng/mL vs. 6.84 [1.48–10.26] ng/mL, *P* < 0.001) but not in the high-grade liver steatosis group (median [IQR], 10.97 [5.13–20.51] ng/mL vs. 6.84 [1.48–10.26] ng/mL, *P* = 0.435) (Figure 1C).

### Identification of Independent Predictors for Liver Steatosis Grading in Obese and Overweight Children

Variables associated with liver steatosis grade (NAFLD scores) were first assessed by univariate analysis. Considering that the levels of FGF-21, adiponectin, and leptin were not normally distributed, values were logarithmically transformed to continuous variables in the statistical models. As shown in Table 2, BMI and body fat mass, including body fat percentage, trunk fat percentage, and total body fat weight, showed a strong correlation with liver steatosis grade (*B* = 0.400–0.493, *P* < 0.001), whereas the waist-to-hip and waist-to-height ratios showed a moderate correlation with steatosis grade (*B* = 0.252–0.315, *P* < 0.01) (Table 2). All tested biochemical parameters were significantly associated with steatosis grade. Of these, AST, ALT, γ-GT, TG, and total cholesterol showed a strong positive correlation (*B* = 0.354–0.603, *P* < 0.001), HOMA-IR and LDL-C showed a moderate positive correlation (*B* = 0.332–0.395, *P* < 0.01), and HDL-C levels showed an inverse correlation with liver steatosis degree (*B* = −0.308, *P* = 0.003) (Table 2). Moreover, the logarithmically transformed FGF-21 levels were positively and significantly correlated with liver steatosis grade (*B* = 0.516, *P* < 0.001) whereas logarithmically transformed adiponectin (*B* = −0.217, *P* = 0.067) and leptin levels (*B* = 0.003, *P* = 0.978) were not.

**Table 2:** Regression analysis of clinical and laboratory parameters in relation to NAFLD score in obese and overweight children.

	**Univariate regression analysis**			**Multiple linear regression with a stepwise procedure**		
	**Beta**	**95% CI**	***P*-value**	**Beta**	**95% CI**	***P*-value**
**ANTHROPOMETRIC AND BODY COMPOSITION DATA**						
BMI (kg/m^2^)	0.493	0.182–0.362	<0.001	0.174	0.009–0.207	0.033
Waist-hip ratio	0.252	1.206–7.863	0.008			
Waist-height ratio	0.315	3.160–11.708	0.001			
Mean skinfold thickness (cm)	0.048	−0.249–0.415	0.622			
Total body fat (%)	0.429	0.067–0.155	<0.001			
Trunk fat (%)	0.443	0.071–0.158	<0.001			
Total body fat (kg)	0.400	0.061–0.153	<0.001			
Fat-free mass (kg)	0.006	−0.031–0.033	0.952			
Total body water (kg)	0.006	−0.043–0.046	0.949			
Total body muscle (kg)	0.010	−0.051–0.056	0.917			
**BIOCHEMICAL DATA**						
HOMA-IR	0.395	0.096–0.469	0.004			
AST (U/L)	0.583	0.041–0.070	<0.001			
ALT (U/L)	0.580	0.018–0.032	<0.001			
γ-GT (U/L)	0.603	0.074–0.128	<0.001	0.368	0.031–0.088	<0.001
Triglyceride (mg/dL)	0.452	0.010–0.022	<0.001	0.180	0.000–0.013	0.035
Total cholesterol (mg/dL)	0.354	0.013–0.038	<0.001			
HDL-C (mg/dL)	−0.308	−0.100 to −0.022	0.003			
LDL-C (mg/dL)	0.332	0.010–0.042	0.001			
**POTENTIAL BIOMARKERS**						
Log FGF-21 (pg/mL)	0.516	1.622–3.213	<0.001	0.273	0.510–2.099	0.002
Log adiponectin (ug/mL)	−0.217	−2.576–0.101	0.067			
Log leptin (ng/mL)	0.003	−1.060–1.089	0.978			

*P-value based on logistic regression analysis; P-values < 0.05 were considered statistically significant; CI, Confidence interval*.

We next used multivariate regression analysis with a stepwise procedure to assess the best predictors for differentiating liver steatosis severity in obese and overweight children (Table 2). The results indicated that stepwise increments in BMI (*B* = 0.174, *P* = 0.033), γ-GT (*B* = 0.368, *P* < 0.001), TG (*B* = 0.180, *P* = 0.035), and log-transformed FGF-21 levels (*B* = 0.273, *P* = 0.002) were significantly correlated the steatosis grade of obese and overweight children (*P* < 0.05) (Table 2).

### Evaluation of Accuracy of Single or Combined Tests to Predict High-Grade Liver Steatosis

**Figure 2:**
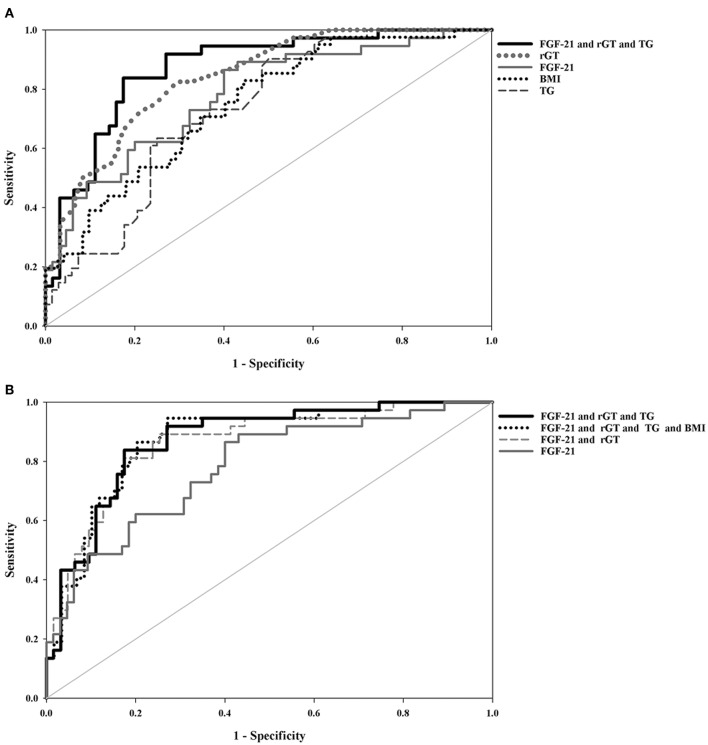
Comparison of area under the receiver-operating characteristic(AUROC) curves between **(A)** single blood test and a combined biomarker panel **(B)** combined biomarker panels and FGF-21 for the prediction of high-grade liver steatosis in the obese and overweight subjects. All values were logarithmically transformed in the statistical models.

As shown in Table 3 and Figure 2, the ROC curve analysis of high-grade liver steatosis in obese and overweight children revealed that γ-GT ≥ 21.50 U/L, FGF-21 ≥ 106.10 pg/mL, TG ≥ 77.00 mg/dL, and BMI ≥ 25.83 kg/m^2^ were significant predictors with optimal AUROC (0.732–0.840) and good sensitivity (82.5–90.24%); nevertheless, the specificity was relatively lower (50.0–70.50%). Of these four indices, γ-GT was the best predictor with an AUROC of 0.840 (95% CI, 0.765–0.915), sensitivity of 82.50%, and specificity of 70.50%. Our further analysis found that the combination of each biomarker with FGF-21 demonstrated better accuracy with an AUROC of 0.861–0.873 and higher specificity of 72.88–82.54% than a single test. Overall, the best performance was obtained by combing FGF-21, γ-GT, and TG with the AUROC of 0.871, specificity of 82.54%, and sensitivity of 83.78% for predicting high-grade liver steatosis (Figure 2B).

**Table 3:** Summary receiver's operating characteristic (ROC) of variables in the prediction of high-grade liver steatosis in the obese and overweight subjects.

**Single blood test**	**AUROC**	**Cut-off value**	**95% CI**	**Sensitivity%**	**Specificity%**	***P*-value**
γ-GT (U/L)	0.840	21.50	0.765–0.915	82.50	70.50	<0.001
FGF-21 (pg/mL)	0.781	106.10	0.687–0.874	86.49	60.00	<0.001
BMI (kg/m^2^)	0.748	25.83	0.657–0.839	82.93	55.56	<0.001
TG (mg/dL)	0.732	77.00	0.639–0.824	90.24	50.00	<0.001
**Combined tests**	**AUROC**	**Cut-off value (Log)**	**95% CI**	**Sensitivity%**	**Specificity%**	***P*****-value**
FGF-21 and γ-GT	0.861	3.318	0.786–0.937	89.19	74.60	<0.001
FGF-21 and γ-GT and TG	0.871	5.403	0.801–0.942	83.78	82.54	<0.001
FGF-21 and γ-GT and TG and BMI	0.873	6.661	0.801–0.945	94.59	72.88	<0.001

*TG, triglyceride; CI, confidence interval*.

*P-value based on ROC. P-values < 0.05 were considered statistically significant*.

*Combined tests: all values were logarithmically transformed in the statistical models*.

## Discussion

In this study, we demonstrated that γ-GT, FGF-21, TG, and BMI were significantly associated with liver steatosis severity in obese and overweight children and may be used as the initial assessment. In addition, the combination of FGF-21 with these biochemical parameters can further improve the accuracy for predicting high-grade liver steatosis than either test used alone. Overall, the combination of FGF-21, γ-GT, and TG showed the best performance. Taken together, our results support the potential role of FGF-21 in pediatric NAFLD.

Recently, relationships between higher FGF-21 levels and adverse lipid profiles, obesity, metabolic syndrome, or type 2 diabetes mellitus have been reported in adults (24). However, the role of FGF-21 in the development of NAFLD is not well-understood. Evidence from studies in adults show that elevated FGF-21 levels reduce hepatic lipogenesis and improve insulin sensitivity (25, 26) and are negatively correlated with the probability of NAFLD and NASH (14). Nonetheless, several studies of pediatric patients, including our own, seem to contradict these results (13, 15, 17). In an ultrasonography-based 3-years prospective study, Li et al. reported that children who progressed to NAFLD had significantly higher baseline and follow-up FGF-21 levels than those who did not, suggesting that baseline FGF-21 level is an independent predictor of NAFLD (27). In another pediatric study, Giannini et al. reported that FGF-21 levels were increased in obese children with high hepatic fat content in MRI and significantly correlated with the NAFLD severity score in liver specimens (28). A possible explanation for the increase in FGF-21 concentration in our subjects with NAFLD may be due to a feedback-induced protective response against hepatic steatosis or an FGF-21-resistant state (15, 26). As measuring FGF-21 levels is simple, quick, and inexpensive, we suggest that FGF-21 can be used as a biomarker for predicting pediatric NAFLD in the future (29).

Our data revealed that no single test had good sensitivity and specificity for predicting liver steatosis severity, whereas combined biomarker panels showed better performance. Among the biochemical parameters, γ-GT was the best predictor for high-grade liver steatosis in our study. As a high γ-GT has been shown to be associated with advanced fibrosis in NAFLD (30), the higher γ-GT in our subjects with high-grade liver steatosis imply the possibility of progressive liver fibrosis (31). Notably, AST and ALT were not identified as independent predictors for liver steatosis grading in the logistic regression model. Previous studies also reported that approximately 80% of NAFLD subjects had normal ALT levels (32), indicating that ALT is not justified as an independent test for screening for high-grade steatosis in children (33).

Increasing evidence indicates that adipose tissue can produce multiple adipokines that trigger inflammatory processes, insulin resistance, and dyslipidemia (7, 24, 34, 35) as well as contribute to pediatric NAFLD and high-grade liver steatosis (18, 35, 36). In this study, an increased BMI showed better discriminative ability for high-grade liver steatosis than other obesity indices (e.g., waist circumference, waist-to-hip ratio, and waist-to-height ratio). BMI is easy to measure; nonetheless, the specificity (55.56%) is insufficient. Thus, the measurement of BMI in combination with the above laboratory parameters is recommended.

The strength of this study lies in our thorough evaluation of different clinical and laboratory parameters and the inclusion of FGF-21 as part of a biomarker panel to predict liver steatosis severity. To the best of our knowledge, only a few pediatric studies have evaluated the discriminative capability of FGF-21 based on its sensitivity and specificity for NAFLD (27, 29), and none of the pediatric studies have combined FGF-21 with other serum biomarkers for predicting liver steatosis severity (29). Abdominal ultrasonography is broadly used as a screening method for hepatic steatosis (37). In the present study, we applied a scoring system to provide detailed information about liver steatosis severity (NAFLD scores, 0–6), which is different from other ultrasound-based studies involving children (10, 12, 15, 16). In addition, the NAFLD scores in each participant were scored by the same pediatric gastroenterologist in an attempt to reduce the bias. The limitations are mainly linked to the cross-sectional nature of the study, and we did not perform liver biopsy in these cases. Therefore, whether FGF21 levels are correlated with the long-term outcome or biopsy-proven NASH stages was not confirmed. Further studies to explore serial samples of FGF-21 levels and the association with histology of fatty liver disease severity and longitudinal outcome may increase the significance of these findings. Furthermore, the study sample size was relatively small, and ultrasound did have limitations for detecting mild liver steatosis, which might have influenced the results. More cases are required to validate our findings, including the BMI, FGF-21, γ-GT, and TG cut-off values for detecting high-grade liver steatosis in obese and overweight children and confirming the role of FGF-21 in clinical practice.

## Conclusion

Our results demonstrated that γ-GT, FGF-21, and TG levels and BMI were strongly correlated with liver steatosis severity. Combining FGF-21 and the above biochemical parameters as a biomarker panel may help identify obese and overweight children at risk of developing high-grade liver steatosis.

## Data Availability Statement

The datasets generated for this study are available on request to the corresponding author.

## Ethics Statement

This study was approved by the Research Ethics Committee of Chang Gung Memory Hospital (104-7100C, 106-3610C, and 104-7145B) and complied with the declaration of Helsinki. Written informed parental consent was obtained.

## Author Contributions

M-CH, J-LH, C-CH, T-CY, and M-WL were involved in the study design, participates recruitment, and written consent. M-CH involved in the laboratory work, statistical analysis, and interpretation of its results. M-CH wrote the first draft of the manuscript. M-WL and T-CY edited the first draft of the manuscript. All authors reviewed the manuscript and approved the final version of the manuscript.

### Conflict of Interest

The authors declare that the research was conducted in the absence of any commercial or financial relationships that could be construed as a potential conflict of interest.
